# Extramedullary progression of multiple myeloma despite concomitant medullary response to multiple combination therapies and autologous transplant: a case report

**DOI:** 10.1186/1752-1947-8-299

**Published:** 2014-09-08

**Authors:** Anup Kasi Loknath Kumar, Christopher Dakhil, Megha Teeka Satyan, Nisreen Haideri

**Affiliations:** 1 Division of Hematology and Oncology, University of Kansas Medical Center, Kansas City, KS, USA; 2 Cancer Center of Kansas, Wichita, KS, USA; 3 Division of Hematology and Oncology, Saint Luke’s Health System, Kansas City, KS, USA

**Keywords:** Extramedullary, Multiple myeloma, Plasmacytoma

## Abstract

**Introduction:**

Extramedullary myeloma that occurs during the clinical course of multiple myeloma is rare but is an independent poor prognostic factor with mortality of 73% and median survival of 12 months despite aggressive therapies including novel agents. The clinicopathological aspects, biology and management of extramedullary myelomas are poorly understood. Our case highlights the pathobiological aspects of this important but rare entity, and the repercussions of modern therapies.

**Case presentation:**

A 60-year-old Caucasian man initially presented with an anterior rib fracture. Subsequent workup revealed stage IIIB immunoglobulin G lambda multiple myeloma. A bone marrow biopsy showed sheets of plasma cells, harboring unfavorable cytogenetics including deletion of 17p and t(4;14). He achieved near complete remission and resolution of karyotypic abnormalities with three cycles of induction doxorubicin, thalidomide, and dexamethasone (clinical trial). This was followed by high-dose melphalan and autologous stem cell transplant. He relapsed 1 year later. His bone marrow at that time showed only a few scattered polyclonal plasma cells. He received three cycles of bortezomib and tanespimycin (clinical trial) and achieved very good partial response. He again relapsed 1 year later with multiple large peripheral soft tissue masses and lymph nodes. Biopsies of the peripheral lesions were consistent with extramedullary myeloma, but repeat bone marrow biopsy continued to show no evidence of intramedullary disease.

**Conclusions:**

This is one of the few cases reported that illustrates the differential response of extramedullary compared to intramedullary myeloma to multiple standard combination therapies including novel therapeutics and transplant, resulting in a very short survival. Several mechanisms for intra-to-extra medullary migration and hence the differential treatment response have been hypothesized. Physicians should be aware of this problem during treatment with immunomodulatory drugs and proteasome inhibitors not only in relapsed but also in front-line setting. In such cases, there is a potential role for evolving targeted therapeutics as we continue to better understand the tumor biology.

## Introduction

Multiple myeloma (MM) is a plasma cell malignancy that remains incurable despite the use of high-dose chemotherapy with autologous stem cell transplantation (ASCT) [1]. It is the second most common hematological malignancy following lymphoma, affecting over 20,000 patients each year in the USA, with nearly 11,000 deaths during the same time period [2]. MM is a disease characterized by neoplastic proliferation of monoclonal plasma cells that lead to lytic bone lesions, hypercalcemia, and renal impairment. Survival of MM may be prolonged with novel agents such as thalidomide, which has been demonstrated to be effective for refractory MM [3]. In addition, the combination of melphalan and prednisolone with thalidomide (MPT) is also an effective first-line treatment for elderly patients with MM [4].

Plasmacytomas rarely arise outside the bone marrow because of the favorable microenvironment in the marrow. Extramedullary plasmacytoma (EMP) is a plasma cell neoplasm of soft tissue without bone marrow involvement and hence no systemic signs and symptoms associated with MM [5]. Therefore they may be incidentally diagnosed on imaging studies obtained for a different indication. They are typically solitary, radiosensitive neoplasms that frequently pursue an indolent clinical course, constituting approximately 3% of all plasma cell tumors with a male to female ratio of 3:1 [6], with a low recurrence rate (<10%) and high 10-year disease-free survival. The disease may involve a wide variety of anatomic sites: upper aerodigestive tract comprising nasal/paranasal, pharynx, trachea and esophagus (85%), pancreas, spleen, urinary bladder, thyroid, breast and testicles [7]. MM preceded by an EMP is seen in approximately 15 to 20% of patients with MM as a manifestation of EMP progression [7,8]. In contrast, extramedullary MM (eMM) that occurs during the clinical course of MM is rare, although its incidence is increasing in recent years (Table 1) [1,8], approximately 14% per the Spanish Registry of Transplants [9]. eMM is an independent poor prognostic factor, with mortality of 73% and median survival of 12 months despite aggressive therapies [10]. Response to immunomodulatory drugs (IMiDs) in patients with eMM is poor [1,11]. Here we present an extremely uncommon case of progressive multiple eMM despite concomitant medullary response after multiple combination therapies and ASCT. The case reported here highlights the pathobiological aspects of this important but rare entity, and the repercussions of modern therapies.

**Table 1:** List of cases with extramedullary multiple myeloma progression despite concomitant medullary response to multiple myeloma therapy

**Case study/series**	**Number of cases**	**Multiple myeloma treatment**	**Bone marrow at extramedullary multiple myeloma relapse**	**Extramedullary multiple myeloma relapse course/response**
Bairey *et al*. [12]	1	Chemotherapy	No evidence of disease	eMM relapse in skin and subcutaneous areas, right eyebrow, right knee, sternum and right axilla. Failed chemotherapy. Died secondary to liver eMM.
Iwasaki *et al*. [13]	1	Chemotherapy	No evidence of disease	Skin eMM relapse in 6 months which responded to chemotherapy. Retroperitoneal eMM relapse 2 years later causing death.
Avigdor *et al*. [11]	2	Chemotherapy followed by ASCT. Relapse of MM was treated with thalidomide.	No evidence of disease	Patient 1 developed parasellar eMM after 3 months of thalidomide and died in 2 weeks. Patient 2 developed diffuse skin eMM which failed to respond to Allo SCT.
Ah-Weng *et al.*[14]	1	Chemotherapy followed by ASCT	No evidence of disease	Multiple cutaneous eMM in 3 months. VAD salvage chemotherapy followed by localized RT and IFN-2a attempted but patient died in 2 weeks.
Terpos *et al*. [10]	15	ASCT or Allo SCT	No evidence of disease	median time from ASCT to eMM was 24 months. eMM sites included skin, rectum, and testicles. Treated with local RT (n=5), combination of RT and chemotherapy or thalidomide (n=7), and chemotherapy +/- thalidomide (n=2), VAD-chemotherapy and local RT followed by a mini-allograft from the original donor (n=1). 11 patients died at a median of 10 months following diagnosis of eMM. 4 patients were still alive at 12–20 months after eMM relapse.
Candoni *et al*. [15]	3	Thalidomide	No evidence of disease	Median time to eMM relapse was 3 months. eMM sites included cutaneous, soft tissue, parasellar. Salvage therapy attempted but poor clinical outcome.
Cerny *et al*. [16]	6	IMiD and/or ASCT	n/a	Median time to progression and survival after eMM relapse was 29 months and 38 days respectively.
Waterhouse *et al*. [17]	1	Thalidomide/melphalan followed by ASCT. MM relapse treated with bortezomib.	No evidence of disease	eMM relapse in the brain, pleural and paravertebral soft tissue in 1 month following bortezomib.
Gozzetti *et al.*[18]	1	Chemotherapy followed by ASCT	No evidence of disease	eMM relapse in lung, mediastinum, pancreas, psoas muscle at 5 months post-ASCT. Failed hyper C-PAD. Disease stabilized on lenalidomide at 10 months from eMM relapse.

Abbreviations: Allo SCT, allogeneic hematopoietic stem cell transplantation; ASCT, autologous stem cell transplantation; eMM, extramedullary multiple myeloma; IFN, interferon; IMiD, immunomodulatory drug; MM, multiple myeloma; n/a, not available; VAD, vincristine, doxorubicin, and dexamethasone; RT, radiation therapy; C-PAD, cyclophosphamide, liposomal pegylated doxorubicin, bortezomib and dexamethasone.

## Case presentation

**Figure 1:**
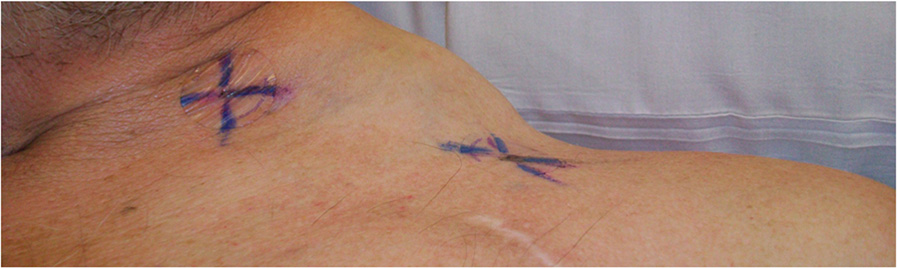
Photograph of patient’s left supraclavicular group of lymph nodes.

**Figure 2:**
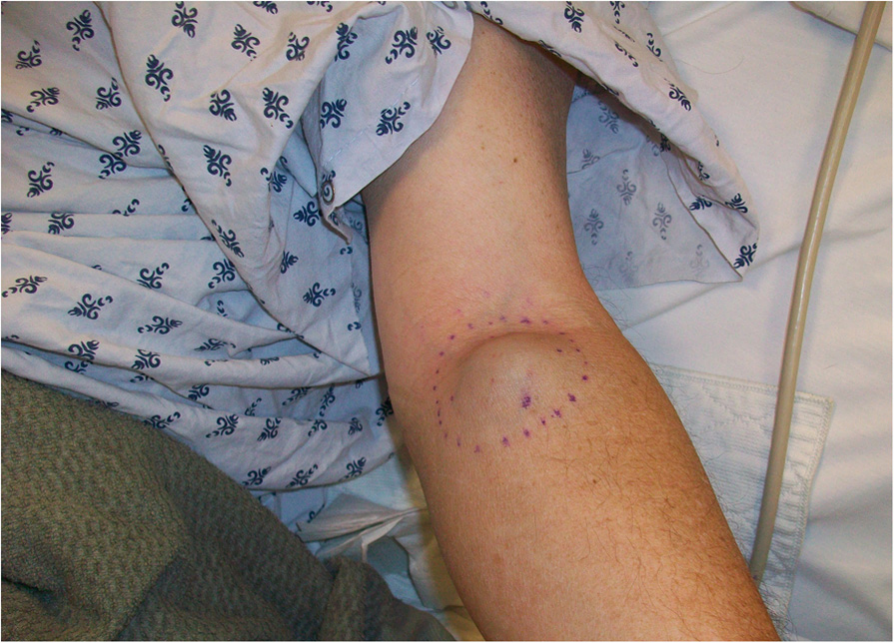
Photograph of patient’s soft tissue mass in the left antecubital fossa.

**Figure 3:**
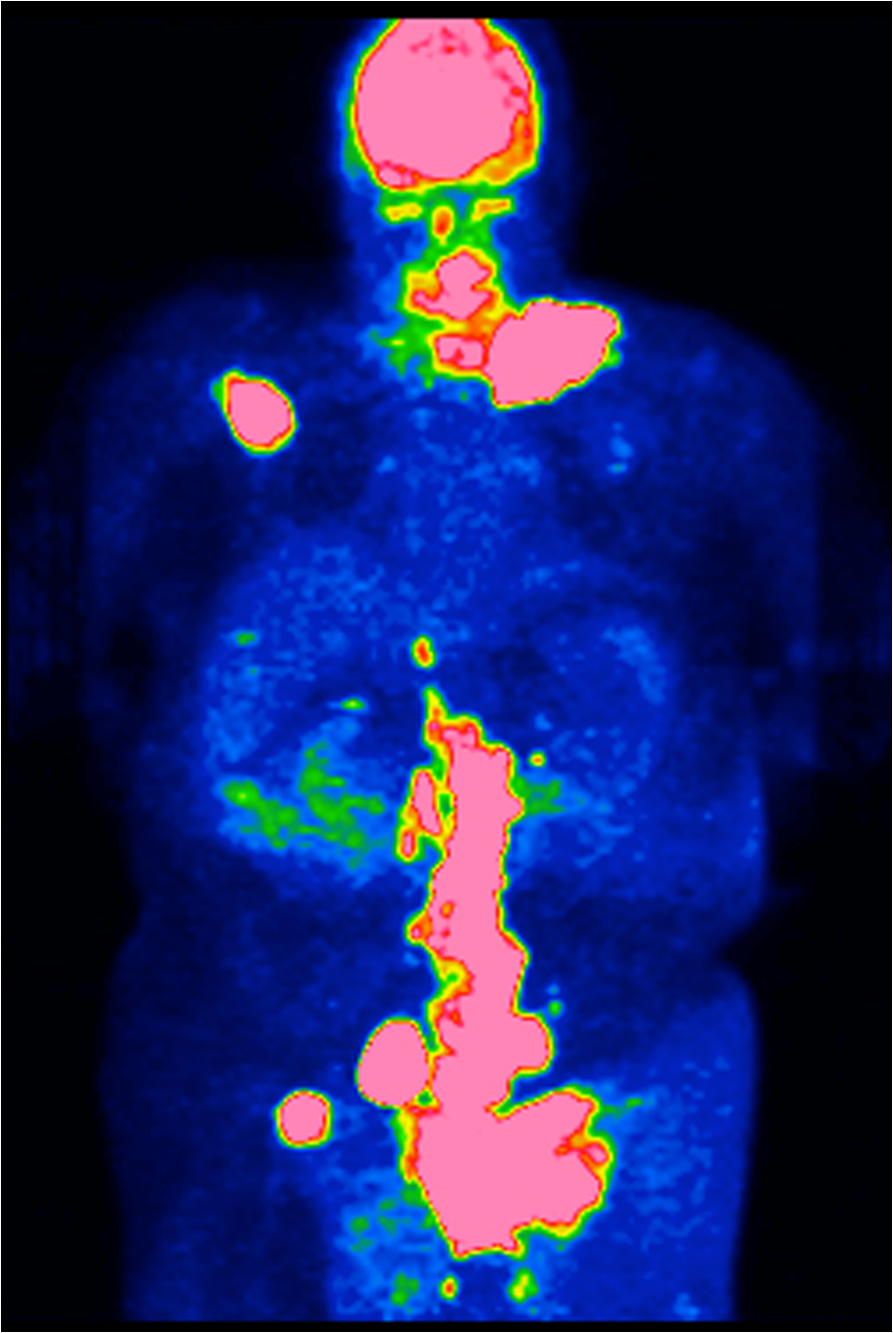
Positron emission tomography scan showing metabolic activity in the enlarged left supraclavicular, inguinal and abdominal lymph node regions.

**Figure 4:**
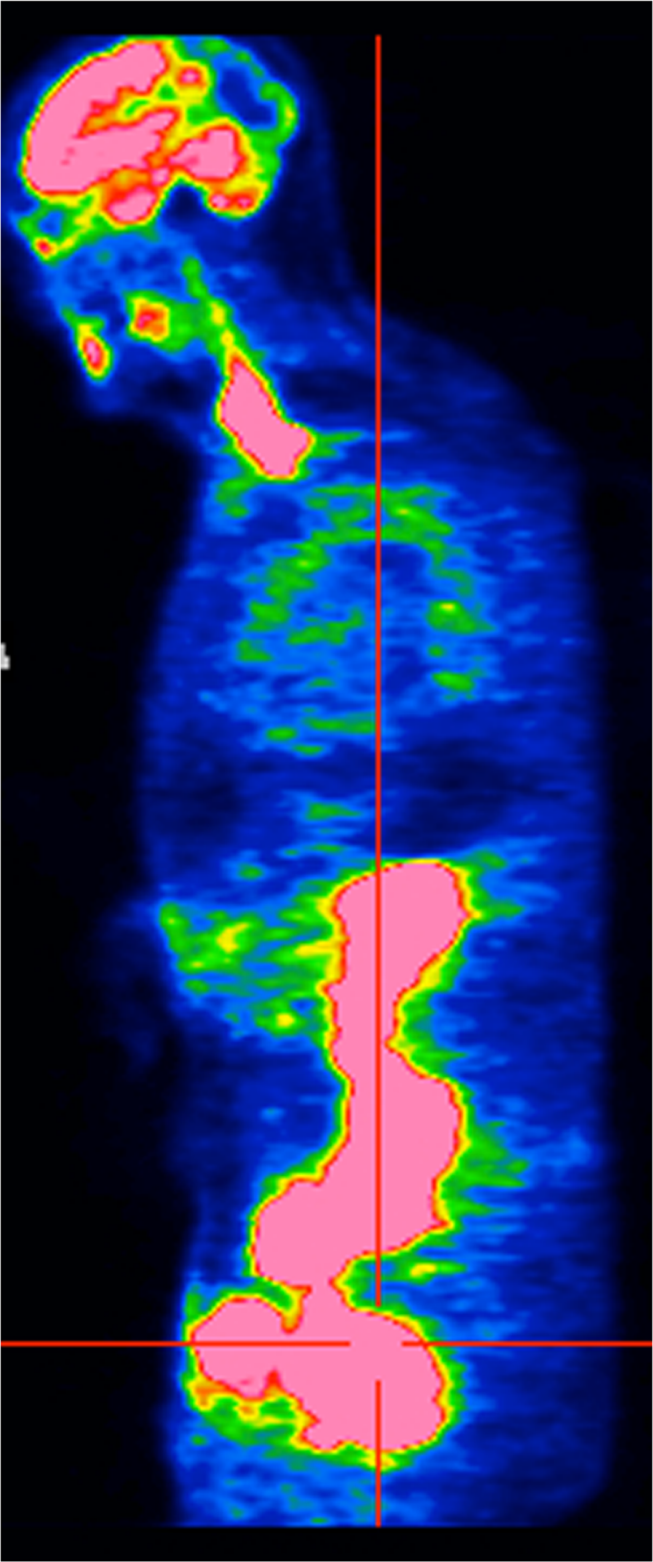
Positron emission tomography scan showing metabolic activity in the numerous intra-abdominal and left inguinal soft tissue masses.

A 60-year-old Caucasian man initially presented with an anterior rib fracture which was evaluated with X-rays that revealed lytic lesions. Subsequent workup for MM revealed elevated immunoglobulin (Ig) G levels and masses in his anterior rib that appeared to be plasmacytomas. The results of initial laboratory tests revealed an IgG lambda paraprotein, serum protein electrophoresis 6.36g/dL, urine protein electrophoresis 1956mg/24 hours, IgG of 8000, lambda free light chain of 151, beta-2 microglobulin of 10.8, hemoglobin 9.3g/dL and serum calcium of 14.4mg/dL. A skeletal survey showed involvement of disease with multiple lytic lesions in his skull, thoracic spine, ribs, clavicle, pelvis, and bilateral upper and lower extremities. The diagnosis was confirmed by a bone marrow biopsy, which showed sheets of plasma cells. Cytogenetics showed hyperdiploidy, trisomy 5 and 9, deletion of 17p, IgH translocation t(4;14), but were negative for deletion 13q. He was staged as IIIB according to the Durie-Salmon staging system. He was started on induction treatment with a clinical trial using doxorubicin, thalidomide, and dexamethasone. He received three cycles with resultant near complete remission and resolution of karyotypic abnormalities. Five months after induction, he underwent treatment with high-dose melphalan, followed by ASCT. He did well initially after transplant but relapsed approximately 1 year later. Bone marrow at that time showed only few scattered polyclonal plasma cells. He was started on treatment with bortezomib and tanespimycin as part of the University of Kansas Medical Center Institutional Review Board (IRB)-approved clinical trial after having obtained the patient’s consent. He achieved a very good partial response after three cycles. Following the third cycle he was taken off the study for failure to tolerate tanespimycin. He did well for approximately 1 year, but relapsed again with multiple large peripheral lesions including his left supraclavicular lymph nodes (Figure 1), left antecubital fossa (Figure 2), and left inguinal area. Positron emission testing revealed numerous soft tissue masses as shown in Figures 3 and 4. Biopsies of the peripheral lesions were consistent with eMM, but a repeat bone marrow biopsy continued to show no evidence of intramedullary disease. He received radiation to above peripheral eMM with improvement in size and symptoms. Following this he declined further treatment and was placed on palliative care and expired soon after. To the best of our knowledge, this is one of the few cases reported of a patient with rapid extramedullary relapse and progression of disease despite concomitant medullary response to multiple standard combination therapies including novel therapeutics and ASCT.

## Discussion

This rare case of myeloma had several unusual features including unfavorable cytogenetics, progressive eMM despite concomitant bone marrow response to therapy and very rapid progression of disease within 12 months of eMM relapse.

Due to the rarity and variable presentation of EMP, guidelines are unclear for optimal management of this entity but localized palliative radiotherapy is favored for symptomatic lesions [19]. Our patient underwent radiotherapy with improvement in size and symptoms. However, in recent times, radiotherapy has been outmoded by bortezomib, a proteasome inhibitor of NF-KB, as the treatment of choice for eMM due to encouraging outcomes (<10% local recurrence rate) [1,19,20]. Our patient did relapse with multiple EMPs within 12 months of receiving bortezomib. Such increased frequency of eMM relapse has been reported in the past (Table 1), hence suggesting a possible causative relation to current MM therapies.

Thalidomide and lenalidomide (IMiDs) have been relatively recently introduced in the frontline therapy of MM; the induction for transplant eligible patients has changed from cytotoxic chemotherapy to a combination of IMiDs with steroids [21]. Although thalidomide alone [1] or in combination [8,15] has shown antimyeloma activity with both a fall in monoclonal paraprotein levels and clearance of marrow plasmacytosis, its activity in eMM appears variable. Overall, patients with eMM do not appear to attain a good response to IMiDs [1,11,15]. As witnessed in our patient, Candoni *et al*. in 2008 reported three patients with MM but without extramedullary disease at diagnosis, treated with thalidomide plus dexamethasone upfront, who developed eMM during treatment although they were able to achieve fall in monoclonal paraprotein levels and clearance of marrow plasmacytosis [15]. Terpos *et al*. [10] described no definitive advantage by comparing different treatment strategies for eMM recurrence, including local radiotherapy and/or chemotherapy, followed with or without a second ASCT (Table 1). The median time from ASCT to relapse of EMP was 24 months (compared to 12 months in our patient).

Although it remains to be clearly elucidated, several mechanisms have been postulated to explain this differential response. IMiDs via their antiangiogenic and immunomodulating properties can cause alteration in production of interleukin-6 and tumor necrosis factor-a, and decreased adhesion of neoplastic plasma cells. This loss of cell-to-cell interactions within the bone marrow microenvironment may lead to metastasis of malignant plasma cells to extramedullary sites resulting in eMM. This phenomenon indicates that eMM cells have the ability to grow in the absence of bone marrow milieu and hence circumvent antiangiogenic or immunomodulatory effects of IMiDs [21]. Loss of CD56 or lack of CD56 (neural cell adhesion molecule) expression on the primary MM cells appeared to promote eMM recurrence with more aggressive histology and higher proliferation index [16]. In addition, mutations in *K-Ras*[22] and difference in chemokine receptor profiles including functionally intact CXCR4 [23] have been linked to transitioning myeloma cells from intramedullary to extramedullary sites. Moreover, thalidomide requires a bone marrow microenviroment for better antimyeloma efficacy and hence explains its poor activity in eMM [24]. Also, there is difference in the microvascular supply between bone marrow and eMM. In addition the plasma cells may dedifferentiate during thalidomide treatment changing phenotypes that promote drug resistance [25]. Hence, the newer drugs are unable to overcome the impact of this aspect of disease biology and ultimately the condition becomes resistant to salvage chemotherapy [10]. The pathogenesis of eMM post-ASCT is uncertain but may involve re-seeding of myeloma cells (by infusion of stem cells contaminated with MM cells during harvesting) and metastasis of residual myeloma due to incomplete myeloablation [14].

Among the new drugs, bortezomib has been shown to have some effect in patients with eMM, in combination with thalidomide and dexamethasone as first-line treatment [20], and alone or in combination with chemotherapy in patients with prior IMiDs exposure [26]. Although our patient received bortezomib and tanespimycin after treatment with thalidomide/dexamethasone and ASCT, his eMM relapsed suggesting development of extensive chemoresistance as supported by previous reports [10,17]. Preconditioning with high-dose chemotherapy could allow myeloma cells to escape from marrow and subclinical metastases of an extramedullary clone with a high degree of chemoresistance [10]. Similarly, a high incidence of resistant eMM is reported in reduced-intensity conditioning allogeneic transplant [10,27], including patients with chronic graft-versus-host disease (GVHD). Chronic GVHD is complemented by graft versus myeloma effect at the bone marrow level, but at eMM sites, the myeloma cells are able to circumvent this immune-mediated effect in the absence of a bone marrow microenvironment [27]. In these cases of highly resistant eMMs, there is a potential role for evolving therapeutics, such as histone deacetylase (HDAC) inhibitors, heat shock protein 90 (Hsp90) inhibitors, and the alkylphospholipid (Akt) inhibitor [28].

In the post-genomic era, certain cytogenetics are associated with unfavorable prognosis and short survival such as IgH translocation, for example t(4;14), deletions of chromosomes 13 and 17 and abnormalities of chromosome 1 (1p deletion and 1q amplification) [29]. The expression of p53 (17p13) was more prevalent in eMM than in MM [30]. Many of these abnormal cytogenetics were present in our patient including del(17p), for which effective therapy is unavailable based on current evidence. Accumulation of genetic and epigenetic aberrations may lead to morphological progression and probably are accompanied by biologically aggressive eMM [21].

In the clinical world, due to the rarity, unusual locations and high-grade histology, eMM can pose a great diagnostic challenge to treating physicians and pathologists. The differential diagnosis in our case included a poorly differentiated carcinoma, metastatic carcinoma, melanoma, various lymphomas or high-grade sarcoma. Without knowledge of prior history, the initial workup may not reveal the diagnosis even after employing immunohistochemical stains. An accurate diagnosis requires increased awareness of eMM, a good clinicopathological interaction, and a precise application of plasma cell markers (CD138, CD38, kappa, lambda light chain) by immunohistochemical stains.

## Conclusions

Progressive eMM despite medullary response after receiving multiple standard combination therapies including novel therapeutics and ASCT is an uncommon presentation of relapsed disease, although its incidence has been on the rise in recent years due to the new therapeutic paradigm in patients with MM. Several mechanisms for intra-to-extra medullary migration and hence the differential treatment response have been hypothesized as reviewed above. Patients with MM who develop eMM often show an increased lactate dehydrogenase, resistance to conventional chemotherapy or immunomodulatory treatment, and a very short survival. Tumor biology of eMM is different from the medullary disease, and MM under IMiD therapy may show extramedullary progression independent of medullary disease regression. Although promising in treating extensive plasmacytosis of bone marrow, bortezomib may not be effective for treating all eMM relapses, or a highly chemoresistant clone. In such cases, there is a potential role for evolving therapeutics, such as HDAC inhibitors, Hsp90 inhibitors and Akt inhibitors.

## Consent

Written informed consent from our deceased patient's next-of-kin for publication could not be obtained despite all reasonable attempts. Every effort has been made to protect the identity of our patient and there is no reason to believe that our patient would have objected to publication.

## Abbreviations

Akt: Alkylphospholipid; ASCT: Autologous stem cell transplantation; eMM: Extramedullary multiple myeloma; EMP: Extramedullary plasmacytoma; GVHD: Graft-versus-host disease; HDAC: Histone deacetylase; Hsp90: Heat shock protein 90; Ig: Immunoglobulin; IMiD: Immunomodulatory drugs; MM: Multiple myeloma; RT: Radiation therapy.

## Competing interests

The authors declare that they have no competing interests.

## Authors’ contributions

All authors contributed to the writing of the manuscript. All authors read and approved the final manuscript.
